# Study on the Pore Structure of Lightweight Mortar with Nano-Additives

**DOI:** 10.3390/nano13222942

**Published:** 2023-11-14

**Authors:** Yiying Du, Ina Pundienė, Jolanta Pranckevičienė, Aleksejs Zujevs, Aleksandrs Korjakins

**Affiliations:** 1 Laboratory of Concrete Technology, Institute of Building Materials, Vilnius Gediminas Technical University, Linkmenų Str. 28, LT-08217 Vilnius, Lithuania; yiying.du@vilniustech.lt (Y.D.); jolanta.pranckeviciene@vilniustech.lt (J.P.); 2 Department of Building Materials and Products, Faculty of Civil Engineering, Riga Technical University, Kipsalas St. 6A, LV-1048 Riga, Latvia; aleksejs.zujevs@gmail.com (A.Z.); aleksandrs.korjakins@rtu.lv (A.K.)

**Keywords:** fly ash cenospheres, lightweight mortar, nano-additives, pore structure, strength

## Abstract

With the development of nanotechnology, nanomaterials have been introduced to improve the engineering properties of cement-based building materials. An abundant number of studies have been carried out on normal-weight concrete using multi-walled carbon nanotubes (MWCNTs) or nano-silica (NS) and have proven their effectiveness. Nevertheless, still very few investigations are available in terms of lightweight cement-based materials, especially when MWCNTs and NS are binarily incorporated. Thus, in this study, fly ash cenospheres (FACs) according to cement weight were applied as lightweight fine aggregates to produce lightweight mortar (LWM). MWCNTs at 0.05, 0.15, and 0.45% and NS at 0.2 and 1.0% were binarily added as modifiers. Compressive and flexural strengths were tested to investigate mechanical behaviors. A water absorption test was conducted, together with scanning electron microscopy (SEM) and mercury intrusion porosimetry (MIP), to identify the impacts of the nano-additives on the pore structure of LWM. The following results were obtained: MWCNTs and NS demonstrated synergic effects on enhancing the mechanical properties of LWM. MWCNTs exerted positive impacts on reducing the porosity and improving the pore distribution at low dosages of 0.05 and 0.15%. The hybrid addition of NS further transformed large voids into small ones and introduced closed pores.

## 1. Introduction

The application of nanotechnology in concrete traces back to the early millennium along with the appearance of growing demands in creating ultra-high-performance concrete [1]. The meaning of nanotechnology is usually defined as “the understanding, control, and restructuring of matter on the order of nanometers (i.e., less than 100 nm) to create materials with fundamentally new properties and functions” [2]. Nanoconcrete is referred to as “concrete that utilizes nanomaterials or concrete with nanomaterials added in which the size of the nanoparticles is less than 500 nm” [1]. The utilization of nanotechnology in concrete materials is divided into two avenues, nanoscience and nanoengineering [3,4]. The latter is a rapidly emerging method that is used to modify concrete materials and is usually fulfilled by the incorporation of nanoparticles and nanotubes. Nano-sized additives have a high surface energy and chemical activity, strongly influencing the formation of structure boundary layers in the mineral matrix.

NS and MWCNTs are the two most widely applied nanoparticles in building materials. NS can promote the hydration process and participate in the pozzolanic reaction with calcium hydroxide (CH), generating more CSH gel, which can fill the tiny pores in the cement matrix [5,6]. The filling effects of NS can reduce the porosity of cementitious materials [7]. MWCNTs are tube-shaped structures consisting of one-atom-thick rolled carbon sheets, with the chemical bonding of the carbon atoms entirely constituted by sp2 bonds, which endow MWCNTs with a high Young’s modulus and strength [8]. MWCNTs can be classified into single-walled carbon nanotubes (SWCNTs) and MWCNTs. In most research works, MWCNTs are used over SWCNTs because they are of lower cost and demonstrate a relatively lower tendency to agglomerate, benefiting the production of better-quality suspensions [9]. Due to the crack-bridging effects and filling mechanisms of MWCNTs, they are promising materials for improving the resistance against crack propagation and enhancing the mechanical performance of concrete [8,10,11]. In addition, MWCNTs also show the ability to facilitate the process of cement hydration and improve the microstructure of hydration products, which contributes to the melioration of the pore structure and macro-properties of concrete [12,13]. 

Regarding the study of NS, an abundant number of investigations have been conducted to research its single effects on cement-based materials. The focus has been placed on the mechanical and physical properties and the microstructure of cement-based materials, accompanied by discussions to reveal the mechanism behind them [14,15,16,17,18,19]. In other efforts, some researchers studied the synergic effects of NS together with other additives, such as ground granulated blast-furnace slags, fly ash, and coir fibers, which have been gaining preference among researchers in recent years [20,21,22,23,24,25]. Similarly, in terms of the investigations on MWCNTs, attention has been paid to their effects on the mechanical properties and durability of materials [10,26,27,28]. Some studies examined the synergic effects of MWCNTs together with other additives on cement composites and concrete, such as basalt fibers, silica fume, and graphene oxide [29,30,31,32]. 

However, most attempts were only made on normal-weight cement composites and concrete; with regard to the effects of NS or MWCNTs on lightweight cement-based materials, especially with FACs as lightweight fine aggregates, only a few investigations are available. Xi et al. [33] introduced NS to improve the mechanical properties of LWM with FAC. They tested the compressive strength, tensile stress, and strain capacity, and they discussed the microstructures of the mortar. The results proved that NS had the potential to enhance the mechanical characteristics, decrease the porosity, and refine the pore structure. Hanif et al. [34] investigated the single effects of NS on the mechanical performance of lightweight cement pastes with FAC. They found that the addition of NS increased the compressive strength, as it facilitated the pozzolanic activity of FAC and enabled better bonding among FAC particles in the cement matrix. Najeeb and Mosaberpanah [35] examined the mechanical performance, durability, and microstructure of LWM with the presence of NS. They pointed out that, due to the utilization of NS and FAC mixtures, a better resistance to high temperatures and sulfuric acid intrusion was observed for the mortar. Liu et al. [36] studied ultra-lightweight cement composites with MWCNTs, metakaolin, and polyethene fibers. The outcomes showed that the binary incorporation of MWCNTs and polyethene fiber led to an increase in mechanical strength. Nevertheless, only the single effects of NS or MWCNTs were involved, and the synergic influence of NS-MWCNTs additives on lightweight cement materials with FAC has rarely been investigated in the currently existing research. 

Thus, FACs were used in this study as lightweight fine aggregates, and MWCNTs and NS were binarily added to modify the mechanical strength and microstructure of mortar. The aim was to fabricate LWM with improved physical–mechanical properties. Compressive and flexural strengths were tested to measure the changes in the mechanical performance of LWM. Via a water absorption test, SEM, and MIP, the synergic effects of MWCNTs and NS on the porosity and pore structure of LWM were identified. FAC is a porous material; therefore, the pore structure of LWM significantly influences its properties. The pore size distribution in LWM plays a key role in determining the water absorption capacity and mechanical behavior. The utilization of nano-additives has been proven effective in improving the mechanical strength of cement-based materials; nevertheless, only limited investigations have been carried out on FAC-based lightweight materials with nano-additives. As FAC has a high porosity, and both MWCNTs and NS can modify materials’ pore size distributions, this study on FAC-based LWM with nano-additives can contribute to the development of such materials for building and construction.

## 2. Materials and Methods

### 2.1. Materials

**Figure 1:**
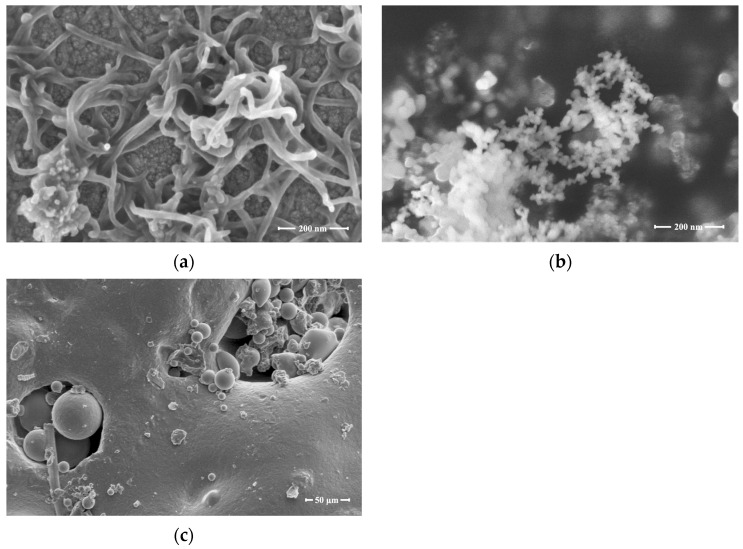
Micrographs of (**a**) MWCNTs; (**b**) NS; (**c**) FAC.

In this study, Portland cement (PC), FAC, MWCNTs, NS, and tape water were used as the main materials. PC was produced by Schwenk according to EN 197-1:2011 [37], and its type was CEM 1 42.5 N. The bulk density of the PC was 0.9 to 1.5 g/cm^3^, and its 28-day standard compressive strength varied from 42.5 to 62.5 MPa. Grade 1 FAC was used as a recycled lightweight fine aggregate. The bulk density of the FAC was 0.37 to 0.40 g/cc, and its size ranged from 40 to 300 μm. Table 1 illustrates the material characteristics of PC and FAC. The MWCNTs used in the study synthesized using the chemical vapor deposition method and NS were binarily utilized to modify the properties of LWM. The diameters of MWCNTs varied between 6 and 15 nm, and their lengths varied from 4 to 13 nm. Figure 1 displays the micrographs of MWCNTs, NS, and FAC under a microscope. In order to obtain a better-quality nanosuspension, surfactant Melment F10 (SP) was added. This is a powdery sulfonated polycondensation product manufactured by BASF Corporation (Ludwigshafen, Germany), and its density was in the range of 500–800 kg/cm^3^.

### 2.2. Mix Design and Preparation of the Samples

Table 2 illustrates the mix ratios and the quantity of each raw material required to prepare 1 cubic meter of LWM. Nine mixes were studied, as well as one control sample with no nano-additives as a comparison. The contents of MWCNTs and NS is a prerequisite for influencing the modifying effects on LWM. Based on the literature [38,39,40,41], inappropriate dosages of nano-additives in cement-based materials cannot improve engineering behaviors and, on the contrary, can even have negative impacts. Hence, the dosages of MWCNTs were determined to be 0.05%, 0.15%, and 0.45% according to the PC weight. And the contents of NS were 0.00%, 0.20%, and 1.00%. The fine aggregate/cement ratio was 73.3%, and the water/cement (w/c) ratio was 56%. The specimen ID Control signifies the control sample; CNT0.05 indicates the sample incorporated with only 0.05% MWCNTs; and CNT0.15NS0.2 refers to the sample with 0.15% MWCNTs, as well as 0.2% NS. The other samples were denominated in the same manner.

**Figure 2:**
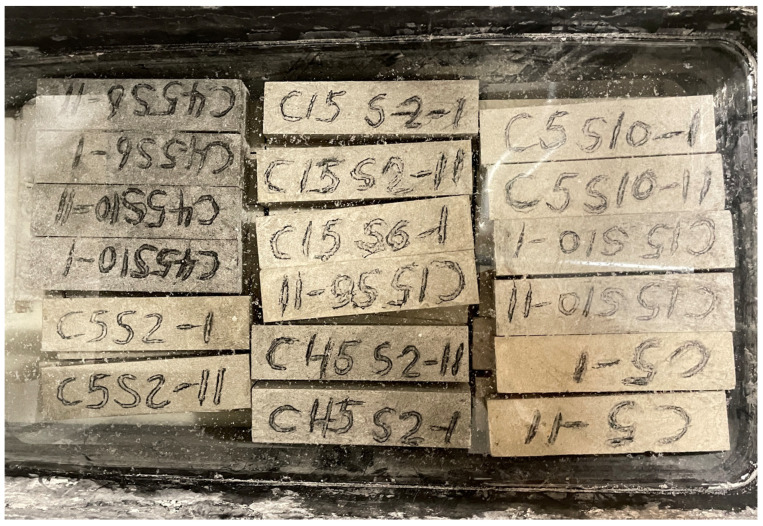
LWM samples cured underwater.

The fabrication of the specimens included two procedures. Initially, a nanosuspension was prepared. Its quality significantly affects the characteristics of modified LWM. The method suggested in most of the literature [26,27,28] was adopted in this study, with the addition of surfactant and the application of ultrasonication. Melment F10 was first weighed and mixed with one-third of the total amount of water. Then, NS and MWCNT powder were added. The solution was evenly stirred and treated with ultrasonic energy with 50 W power and 30 kHz for 30 min. Afterwards, the nanosuspension was mixed into the remaining water and stirred thoroughly for 2 min. The dry mix of PC and FAC was uniformly stirred for 2 min and put into the solution. Then, the mixture was homogeneously stirred using an electronic mixer for 3 min and poured into fully greased metal prism molds in three layers. The size of the prism was 160 × 40 × 40 mm, and a vibration table was used for 2–3 min to guarantee the distribution of the cement mixture. All prisms were then covered with plastic films and placed at room temperature. After 24 h, the samples were demolded and cured underwater in laboratory settings. Figure 2 shows the samples during curing. Lightweight concrete is usually defined as a concrete material with a density between 800 and 2000 kg/m^3^ according to the EN 206-1:2002 standard [42]. The average density of the mortar fabricated in this study was around 1120 kg/m^3^, which falls into the category of LWM.

The workability was examined via a mini-slump flow test on a miniature slump cone of a 60 mm height and a 100 mm bottom diameter. The results showed that, with the absence of NS, the use of MWCNTs effectively improved workability. This is in accordance with the slump flow results obtained in some other studies [10,13,43]. However, after the binary addition of NS, this positive impact was eliminated. Also, better workability was observed in LWM at small dosages of MWCNTs and NS at 0.05 and 0.2%, respectively. Along with the increase in the nano-additive content, the slump flowability declined, especially with the hybrid usage of 0.45% MWCNTs and 1.0% NS, where the worst workability was recorded.

### 2.3. Testing Methods

To determine the mechanical properties, after demolding, the 28-day flexural strength was tested on a WDW-20 universal testing apparatus, and the compressive strength was measured via a 50-C56G2 strength testing machine from CONTROLS.

A water absorption test was performed to investigate the water uptake capacity of LWM by measuring the mass change of the samples. Before the samples were immersed in the water, they were put in an oven at room temperature for drying. The weight of the samples was measured every 24 h. When the weight change was less than 1%, the samples were submerged in the water, and their weights were recorded every 24 h until the 24 h mass change was less than 1%. The characteristics of water absorption were determined based on the mass change due to the absorption of water in contrast with the dry weight of the specimens.

The porosity and pore structure were examined by conducting an MIP test on the samples after the water absorption test. Before the examination, four selected samples (Control, CNT0.05, CNT0.15NS0.2, CNT0.45NS1.0) were oven-dried at 40 °C for 24 h and cut into small cylinders with a cross-section of 6 × 6 mm. The test was performed using a Micro Active AutoPore V 9600 2.03.00 apparatus under the nitrogen ambient with the imposed pressure varying from 0.51 to 59,959.27 psi.

Similar to the MIP test, 4 specimens (Control, CNT0.05, CNT0.15NS0.2, CNT0.45NS1.0) were subjected to the SEM test to qualitatively understand the morphology and microstructure of LWM. A small piece was cracked from the specimen with a hammer and polished to obtain a flat surface before it was observed using a TM 3000 tabletop microscope from Hitachi (Tokyo, Japan).

## 3. Results and Discussion

### 3.1. Flexural Strength

**Figure 3:**
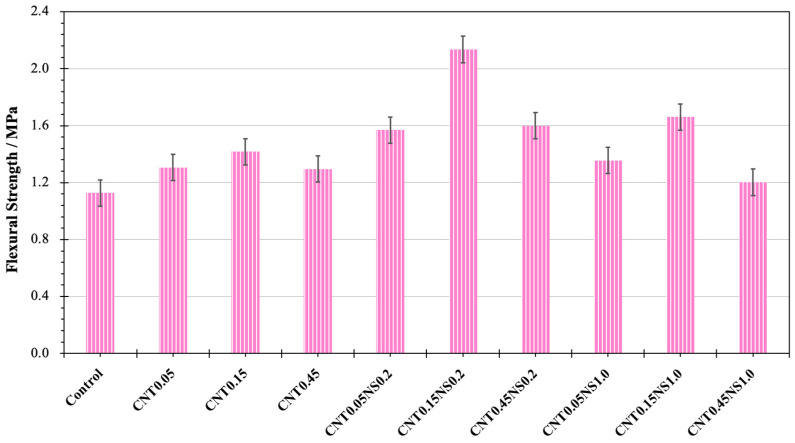
Flexural strength results of LWM.

The flexural strengths of the different LWM samples are depicted in Figure 3. Compared to the Control sample, after the addition of MWCNTs, the flexural strengths all displayed various extents of enhancement. This can be ascribed to the effects of MWCNTs, which can bridge microcracks, introduce more closing pores and prevent the propagation of microcracks in the cement matrix [44,45]. With the hybrid incorporation of NS at the proper dosage, the strength values further improved, indicating that the synergic effects of MWCNTs and NS outperformed the single effects of MWCNTs. This can be associated with the outstanding characteristics of NS that promote the formation of high-density CSH structures and fill the gaps between large particles, effectively improving the microstructure of the mortar [5,7]. The flexural strength of Control was 1.13 MPa. When the content of MWCNTs was 0.15% and the dosage of NS was 0.2%, for specimen CNT0.15NS0.2, the highest flexural strength was recorded to be 2.14 MPa, which was 89.4% higher than that of the Control sample. The smallest increase in strength was measured for sample CNT0.45NS1.0 containing 0.45% MWCNTs and 1.0% NS. The strength was only enhanced by 6.8% to 1.20 MPa.

Regarding the single addition of MWCNTs, by increasing the content of MWCNTs from 0.05 to 0.15%, the flexural strength increased to the peak value of 1.42 MPa, 25.7% higher than the strength of Control. Afterwards, the strength value decreased to 1.29 MPa when the content of MWCNTs increased to 0.45%. With an excess amount, the agglomeration and flocculation of MWCNTs can negatively affect cement hydration and deteriorate the pore structure of cement mortar [46]. After the binary usage of NS, the same evolution of strength was observed at the constant dosages of NS of 0.2 and 1.0%, with the highest strengths (2.14 and 1.66 MPa, respectively) observed when the content of MWCNTs was 0.15%. This demonstrates that, in this study, the optimal dosage of MWCNTs for improving the flexural strength of LWM was 0.15%. Also, with a constant amount of MWCNTs, the specimens containing 0.2% NS all outperformed those containing 1.0% NS, indicating better synergic effects at 0.2% NS. Especially for the specimen incorporated with 0.45% MWCNTs, the binary addition of 1.0% NS led to a 7.0% strength loss compared to the single use of MWCNTs. At a high dosage of 1.0%, excess NS particles can agglomerate and compete with the cement for water, negatively influencing the strength gain [47,48].

### 3.2. Compressive Strength

**Figure 4:**
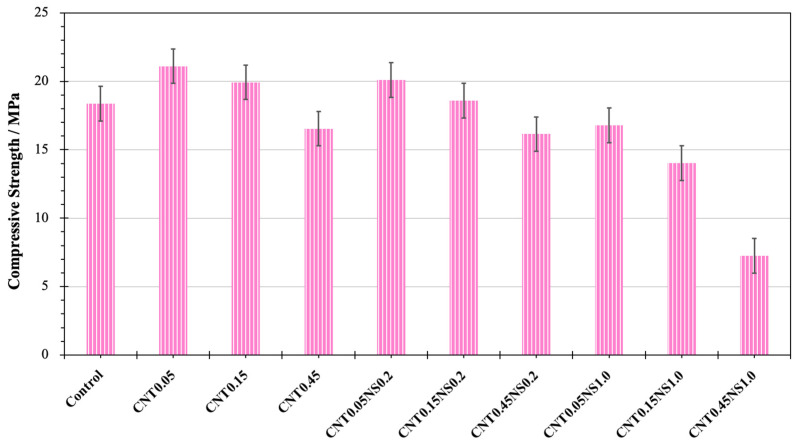
Compressive strength results of LWM.

The results from the compressive strength test are illustrated in Figure 4. The compressive strength of the Control sample was 18.36 MPa, and after the addition of nano-admixtures, both an improvement and a reduction in strength were observed. The highest strength value was recorded for specimen CNT0.05 containing only 0.05% MWCNTs and no NS. The compressive strength was improved by 14.9% to 21.10 MPa. Due to the filler and nucleation effects, MWCNTs can modify the pore structure of LWM and reduce the nano-porosity [12,49]. This is beneficial for strength gain. With the usage of 0.45% MWCNTs and 1.0% NS (sample CNT0.45NS1.0), the lowest compressive strength was measured to be 7.26 MPa, 60.5% less than the strength of the Control sample. The incorporation of high dosages of nano-additives at either 0.45% MWCNTs or 1.0% NS resulted in strength loss. MWCNTs and NS tend to form bundles at high amounts, and the difficulty of achieving a homogeneous dispersion is also increased, which accounts for the formation of the weak zone and the initiation of microcracks, negatively affecting the development of compressive strength [13,46,50].

With the absence of NS, the increase in the MWCNT content from 0.05 to 0.45% contributed to reducing the compressive strength. In contrast with specimen CNT0.05, the strength value of sample CNT0.45 dropped by 21.6% to 16.54 MPa. After the binary addition of NS at 0.2 or 1.0%, the change in the compressive strength with the dosage of MWCNTs exhibited the same pattern. The two highest strength values were both achieved when the amount of MWCNTs was 0.05%, reaching 20.08 and 16.79 MPa respectively. However, as the dosage of MWCNTs was increased to 0.45%, the strength values decreased by 19.6% and 56.8%, respectively. This indicates that 0.05% was the optimal dosage of MWCNTs for the enhancement of compressive strength. At a constant amount of MWCNTs, the increase in the NS dosage led to a reduction in the compressive strength. The samples containing 0.2% NS all displayed better compressive behaviors than the ones with 1.0% NS. According to Karakouzian et al. [51], the high demand for water by NS in cement composites can exert adverse influences on the mechanical properties of samples and affect the extent of the synergic effects of MWCNTs and NS.

### 3.3. Water Absorption

**Figure 5:**
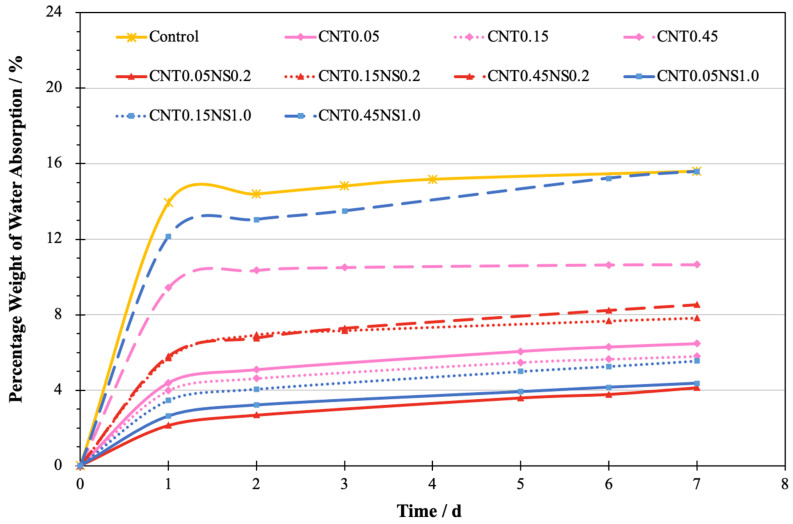
The change of water absorption weight with time.

The water absorption characteristics of cement-based materials refer to their durability and are influenced by the pore structure and microvoids in the hardened materials [52,53]. In Figure 5, the evolution of the water absorbed with the change of time is depicted. During the first 24 h, a sharp increase in the absorbed water amount was observed for all the samples. However, in the following days, the total amount of water absorption only showed a very limited increase. This demonstrates that, at the early stage, the capacity of LWM to absorb water has already achieved saturation. Meanwhile, it was observed that, regardless of the NS content, the weight change curves of the specimens incorporated with 0.45% MWCNTs all possessed a greater velocity than the curves of the other samples with 0.05 or 0.15% MWCNTs. At saturation, they maintained a higher weight of absorbed water. This indicates that lower dosages of MWCNTs at 0.05 or 0.15% are more effective in ameliorating the pore structure of LWM. The reason for this could be associated with the tendency of MWCNTs to agglomerate and flocculate at high amounts, hindering the cement hydration process [46].

**Figure 6:**
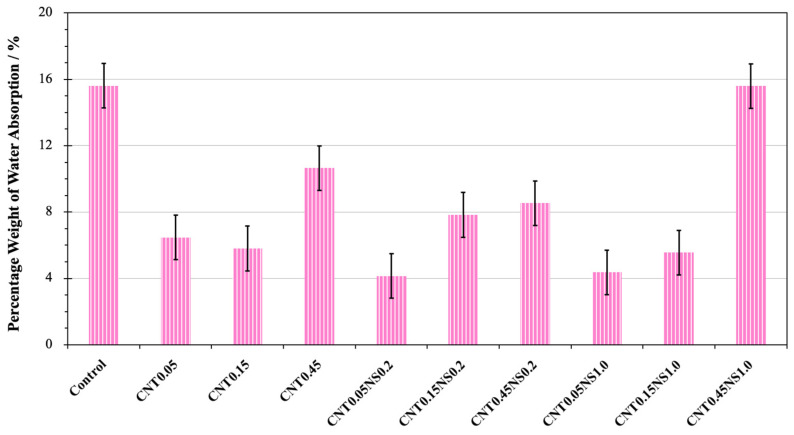
The weight of water absorbed by samples.

Figure 6 displays the percentage weight of absorbed water. This is defined as the difference between the sample weight before and after submersion into the water and normalized by the dry weight of the sample. The Control sample showed 15.6% of water absorption weight. After the addition of nano-additives, the water absorption capacity of all modified samples exhibited different extents of reduction, indicating that the utilization of nano-additives significantly improved the pore structure of LWM. This can be ascribed to the filler effects of MWCNTs and NS, which can fill the micro- and nanovoids in the cement matrix, greatly reducing the porosity [7,49]. Also, MWCNTs can bridge the microcracks and prevent their propagation, which benefits the formation of a denser microstructure in LWM [44,45]. Compared to the Control specimen, the highest decrease in water absorption was measured for sample CNT0.05NS0.2 containing 0.05% MWCNTs and 0.2% NS, with a loss of 11.5% observed; the greatest water absorption capacity of modified LWM was recorded for specimen CNT0.45NS1.0 incorporating 0.45% MWCNTs and 1.0% NS, where 15.6% of water was absorbed (only a reduction of 0.02%). This indicates that a high dosage of the binary addition of MWCNTs and NS is less effective in improving the durability of LWM and ameliorating the microstructure. This can be attributed to the poor dispersion of nano-additives at an excess amount and the high demand for water by NS [50,51]. When the content of NS was constant at 0.0%, along with the increase in the MWCNT dosage from 0.05 to 0.45%, the weight of water absorption showed a downtrend at first, followed by growth. The lowest peak was achieved at 0.15% MWCNTs, with the percentage weight decreasing by 0.67%. For the samples containing 0.2 and 1.0% NS, the percentage of absorbed water weight continued to increase by 4.4 and 11.23%, respectively, as the amount of MWCNTs increased.

### 3.4. SEM Analysis

**Figure 7:**
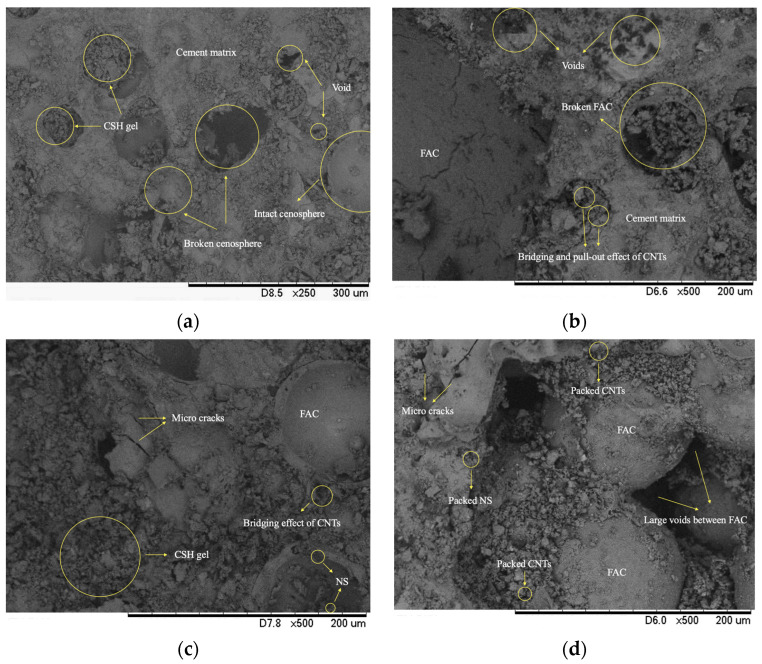
Micrographs of LWM: (**a**) Control; (**b**) CNT0.05; (**c**) CNT0.15NS0.2; (**d**) CNT0.45NS1.0.

To qualitatively analyze the pore structure and morphology, the micrographs of the specimens Control, CNT0.05, CNT0.45NS1.0, and CNT0.15NS0.2 were scanned at the magnifications of 250 and 500, and they are shown in Figure 7. Figure 7a shows the microstructure of the Control sample. Two morphologies of FAC are observed, an intact spherical FAC and a broken FAC. It can be seen that, due to the introduction of FAC, a loose and highly porous microstructure formed. The agglomeration and fraction of FAC enhanced the propagation of large voids in the sample, increasing the porosity of the system and deteriorating the pore structure. Furthermore, the glassy surface and physical–mechanical characteristics of FAC resulted in weak bonds between the aggregates and cement pastes, fostering the formation of microcracks. Although some calcium silicate hydrate (CSH) gels filled the voids and the spaces in the broken FAC, the morphology was still porous and loose, with irregular and non-homogeneously distributed pores.

In Figure 7b, after the addition of MWCNTs, for specimen CNT0.05, a denser and more homogeneous cement matrix was observed, with a smaller number of large voids. The effects of MWCNTs acting the same as fibers to bridge the microgaps and fill the voids were identified, which restrained the propagation of microcracks, significantly improved the pore distribution, and transformed large voids into small ones. However, in the image, a great number of voids can still be seen due to the breakage of FAC, negatively affecting the pore structure. The binary introduction of NS in specimen CNT0.15NS0.2 (Figure 7c) further reduced the number and the size of the voids and microcracks in LWM, owing to its filling effects filling the voids in the cement matrix, forming an increasingly dense microstructure and improving the bond strength of the cement matrix and that between the FAC and the cement matrix. Also, NS can facilitate the hydration reaction of cement, benefiting the formation of more compact CSH pastes. This can effectively optimize the pore structure of LWM.

For specimen CNT0.45NS1.0 in Figure 7d, a more porous and inhomogeneous morphology was observed, with a growing number of large gaps and wide cracks appearing in the cement matrix and between the matrix and fine aggregates. This correlates with the high weight of water absorption and the inferior mechanical performance measured in the tests. At an excess dosage, nano-additives can compete with PC for water [7,47,48]. This hinders the hydration degree and results in incomplete hydration. Furthermore, packed clusters of MWCNTs and NS were observed in the micrographs, demonstrating their poor dispersion in the cement matrix and the formation of agglomeration, deteriorating the pore structure of LWM.

### 3.5. MIP Analysis

**Figure 8:**
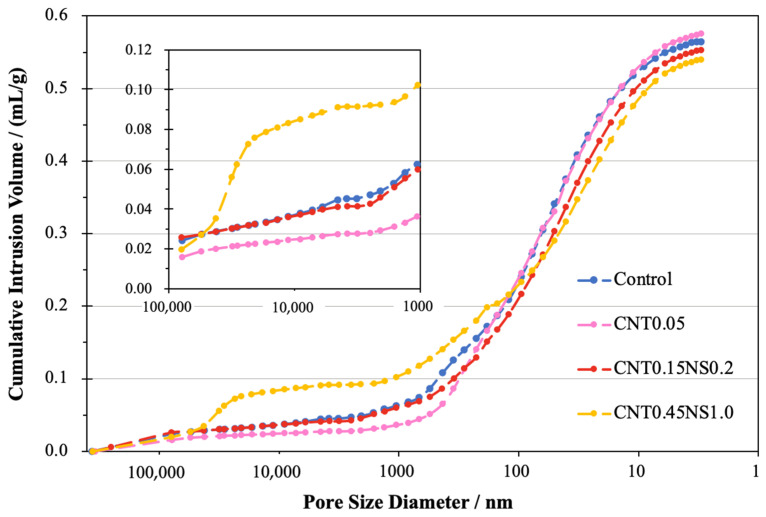
Cumulative intrusion volume of LWM.

To further quantitively evaluate the porosity and pore distribution of LWM under the effects of nano-additives, an MIP test was conducted on four representative specimens (Control, CNT0.05, CNT0.15NS0.2, and CNT0.45NS1.0). In the cumulative intrusion curves displayed in Figure 8, the Control, CNT0.05, and CNT0.15NS0.2 specimens shared similar changing patterns. An obvious sharp enhancement of the total intrusion volume was observed within a pore diameter range from 10 to 1000 nm, indicating the distribution of a large number of voids within this diameter scope. While the evolution of the mercury intrusion curve of sample CNT0.45NS1.0 presented a second steep increase when the diameter of the pores was more than 20,000 nm. And a tremendous increase in the intrusion volume was also observed for pores with a diameter less than 1000 nm. This demonstrates that, in sample CNT0.45NS1.0, an increasing number of large voids existed, which testifies to the results obtained from the morphology graphs and explains its high water absorption and low mechanical strength.

**Figure 9:**
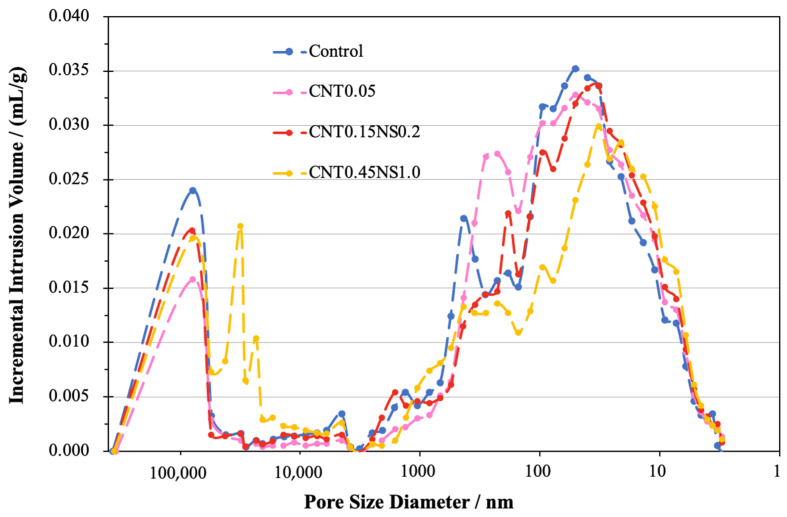
Increase in intrusion volume of LWM.

To compare the overall distribution of the pores in the LWM samples, the derivative volume of intrusion mercury was calculated, and the results are illustrated in Figure 9. Based on the curves, with or without the addition of nano-additives, all four samples exhibited a high intensity of large voids with a diameter of around 100,000 nm. This is associated with the utilization of FAC, which introduces large holes into LWM due to its spherical surface [54]. Another pore size dominating the pore distributions in the Control, CNT0.05, and CNT0.15NS0.2, extended from approximately 6 to 150 nm. Besides these two ranges, the Control sample was also characterized by pores with diameters between 200 and 600 nm. However, specimens CNT0.05 and CNT0.15NS0.2 exhibited peaks of smaller pores mainly from 150 to 500 nm and from 150 to 250 nm, respectively. This demonstrates that, in contrast with the Control sample, the single addition of MWCNTs moved the intensity of the peaks to the left, indicating that the effects of MWCNTs lead to a decrease in large pores and an increase in small pores. This positive impact of nano-additives was further strengthened due to the binary incorporation of NS, with peaks belonging to smaller pores identified in sample CNT0.15NS0.2. The filler effects of nano-additives significantly modified the pore distribution of LWM within the diameter range from 100 to 600 nm, which greatly contributed to the enhancement of its mechanical properties. When the contents of MWCNTs and NS were increased, nonetheless, for specimen CNT0.45NS1.0, the excess addition of nano-additives exhibited a negative influence, contributing to the formation of large voids varying from 21,000 to 55,000 nm. Meanwhile, smaller pores from 7 to 77 nm characterized the pore distribution of CNT0.45NS1.0. This demonstrates that, although the increasing amount of MWCNTs and NS reduced the size of small pores, it accounted for the growth of the tremendous holes formed in LWM, deteriorating the pore structure.

**Figure 10:**
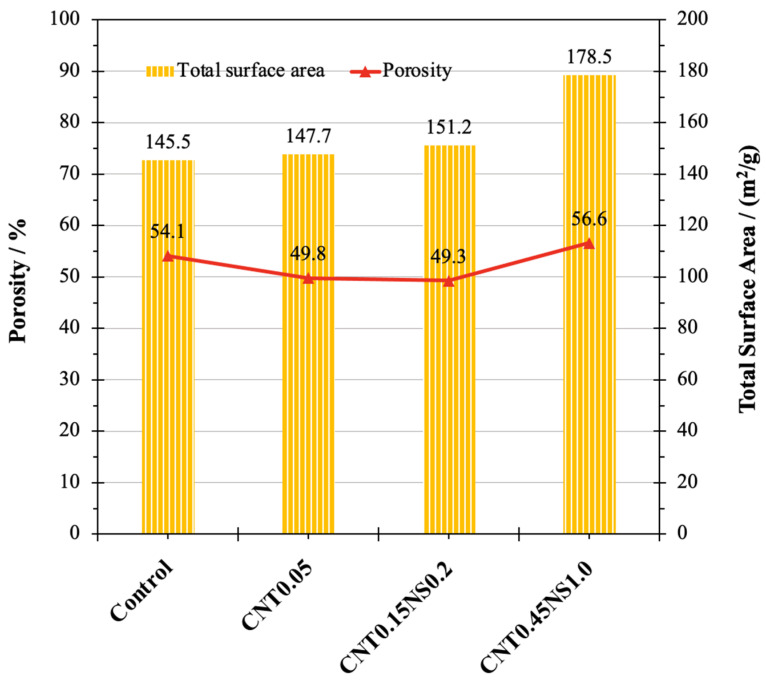
Porosity and total surface area of LWM.

The pore structure of cement-based materials is significantly affected by the porosity and pore size distribution. In Figure 10, the porosity of the representative samples are presented, together with the values of the total surface area of each sample. Along with the addition of MWCNTs and NS at low dosages, the porosity of LWM was significantly decreased. For the Control sample, the porosity was 54.1%, which was decreased by 8.0% to 49.8% with the single incorporation of MWCNTs at 0.05%. When NS was binarily used, the porosity further declined to 49.3%. However, at a high dosage of nano-additives, the porosity of specimen CNT0.45NS1.0 exhibited an increase of 4.6% in contrast with Control, which corresponded to its inferior mechanical performance. In terms of the total surface area, the value for the Control sample was 145.5 m^2^/g, which, after the addition of nano-admixtures, was enhanced by 1.5% and 3.9%, respectively, for samples CNT0.05 and CNT0.15NS0.2. This indicates that the usage of nano-additives contributed to an increasingly complex pore structure of LWM, with a larger number of small pores and a network of interconnecting pores. This can benefit the improvement of mechanical properties by providing more reacting sites for the hydration process. Nonetheless, the large total surface area can be also connected with high porosity, which can weaken the microstructure of samples. This addresses the low strength value and great water absorption of specimen CNT0.45NS1.0, whose surface area increased by 22.8% to 178.5 m^2^/g. 

**Figure 11:**
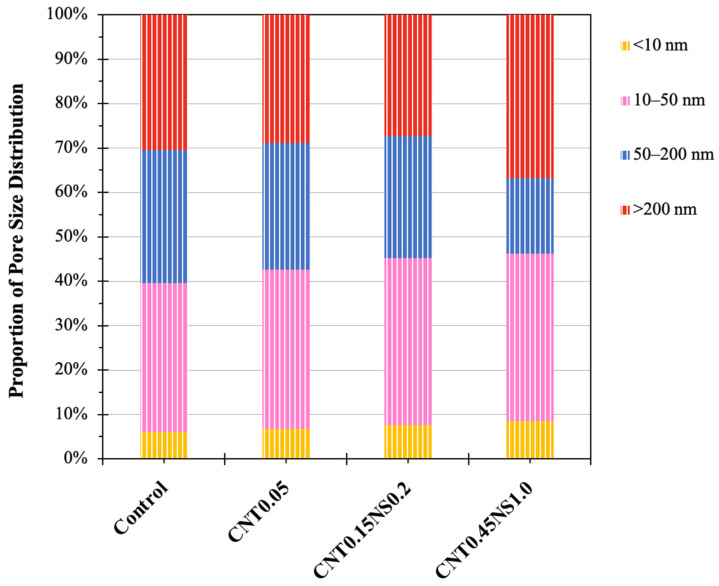
Percentage of pore size distribution of LWM.

The proportions of different pores are presented in Figure 11. Fundamentally, there are three categories of pores in concrete materials, namely, gel pores less than 10 nm, capillary pores between 10 and 200 nm, and macropores larger than 200 nm [28]. Macropores are the big holes that are very harmful to the strength gain of cement materials, and the isolated gel pores existing in hydration products are those that fail to be filled up by hydrates [28,55,56]. Without nano-additives, the Control sample is elementally dominated by capillary pores and macropores, with percentages of 63.46% and 30.50%, respectively. When 0.05% MWCNTs were added alone, the quantity of macropores declined by 5.3%, while the number of capillary pores and gel pores enhanced by 1.3% and 13.3%, respectively. This is associated with the outstanding performance of MWCNTs, which show filler and bridging effects, impressively reducing the number of large pores [8,9]. Also, the incorporation of MWCNTs can facilitate the hydration reaction in LWM, filling the gel pores under 5 nm and resulting in a more compact structure of hydration products [28]. The binary usage of 0.2% NS further improved the number of capillary pores by 2.6% to 65.08% and decreased that of macropores by 10.3% to 27.35% in comparison to the pore distribution of Control. The reduction in large voids and increase in the proportion of small-sized pores in the cement matrix contributed to a denser microstructure and benefited the development of engineering properties. Another point worth noting is that sample CNT0.15NS0.2 possessed a higher percentage of gel pores than CNT0.05, indicating a less dense structure of hydrates, which addresses its higher water absorption results. When 0.45% MWCNTs and 1.0% NS were binarily added, for specimen CNT0.45NS1.0, larger percentages of macrovoids and gel pores were observed, whereas the number of capillary pores reduced to 54.71%. With the high dosage of nano-additives in LWM, the agglomeration of nanomaterials greatly degraded the pore structure of the cement matrix and negatively affected the durability.

According to Fu et al. and Gao et al. [30,55], in cement-based materials, 10–50 nm pores belong to harmless capillaries, while 50–200 nm pores are harmful. In Figure 11, compared to the Control specimen, the addition of nano-additives effectively improved the proportion of harmless capillary pores and reduced that of harmful capillaries, greatly optimizing the pore structure of LWM. And when comparing samples CNT0.15NS0.2 and CNT0.05, the binary usage of MWCNTs and NS exhibited more prominent optimization effects than the single incorporation of only MWCNTs. In specimen CNT0.45NS1.0, although the same optimization effect on the capillary pores was observed, the impressive improvement of the proportion of macropores still deteriorated the pore structure. Generally, high porosity and content of harmful pores are related to low mechanical performance. The results obtained in the MIP analysis are in accordance with the outcomes of the strength tests. Generally, a high porosity and a high content of harmful pores are related to low engineering performance. The results obtained in the MIP analysis are in accordance with the outcomes of the strength and water absorption tests.

## 4. Conclusions

Via compressive strength, flexural strength, water absorption, SEM, and MIP tests, this study investigated the synergic effects of MWCNTs and NS on the mechanical properties, water absorption capacity, pore structure, and microstructure of cement-based LWM with FACs as lightweight aggregates. The results indicate that binary addition greatly contributed to the improvement of mechanical properties, especially flexural behaviors, which benefited from the outstanding tensile behaviors of MWCNTs and their crack-bridging effects. After the addition of 0.15% MWCNTs and 0.2% NS, sample CNT0.15NS0.2 showed the most remarkable flexural strength of 2.14 MPa, 89.4% higher than the strength of the Control specimen. Both an increase and a reduction were observed in the compressive strength. With the absence of NS, the single incorporation of 0.05% MWCNTs (sample CNT0.05) led to the greatest compressive strength of 21.10 MPa, with an increase of 14.9% compared to the strength of Control. 

In the water absorption test, all the samples reached saturation to absorb water in the first 24 h. Afterwards, along with the increase in time, the absorbed water weight was maintained at the same level. Compared to the Control sample with the absence of nano-additives, the utilization of MWCNTs and NS effectively decreased the absorbed water weight to different extents. The most remarkable reduction in water absorption was measured for sample CNT0.05NS1.0. The absorbed water was decreased by 66.28% to 5.8 g. Specimen CNT0.45NS1.0 with a high content of nano-additives exhibited the lowest drop in water absorption by 6.98% to 16.0 g.

In the SEM micrographs, the morphology of the Control sample exhibited a loose microstructure with a great number of large voids due to the introduction of FAC. After the addition of MWCNTs and NS, the microstructure of LWM effectively improved, exhibiting a denser and more homogeneous morphology. However, when an excess amount of nano-additives was used, for specimen CNT0.45NS1.0, the incomplete hydration and agglomeration of nano-additives led to a highly porous microstructure.

Based on MIP, MWCNTs can optimize the pore structure of LWM, and the binary use of NS further improves this effect. Compared to the Control sample, the total surface areas of modified LWM were all improved, and the porosity of CNT0.05 and CNT0.15NS0.2 was reduced by 7.95% and 8.84% respectively. The pore distribution was significantly optimized, with fewer macropores and more capillary and gel pores, which benefits the enhancement of mechanical properties. However, for CNT0.45NS1.0, the incorporation of a high amount of nano-additives led to an increase in porosity of 4.64% and introduced more macropores, deteriorating the pore structure. This resulted in a reduction in the mechanical strength.

## Figures and Tables

**Table 1:** Material characteristics of PC and FAC.

Parameters	PC	FAC
Initial setting time (min)	≥60	-
Size (micron)	-	40–300
Sulfate content (%)	≤3.5	-
Chloride content (%)	≤0.1	-
Bulk density (g/cm^3^)	0.9–1.5	0.37–0.40
2-day compressive strength (MPa)	≥10.0	-
28-day compressive strength (MPa)	42.5–62.5	20–40

**Table 2:** Mix design for 1 m^3^ of LWM.

Specimen	PC (kg)	Water (kg)	FAC (kg)	SP (kg)	MWCNTs (kg)	NS (kg)	MWCNTs (%)	NS (%)
Control	527.34	295.31	386.72	-	-	-	0	-
CNT0.05	527.34	295.31	386.72	2.66	0.27	-	0.05	-
CNT0.15	527.34	295.31	386.72	2.66	0.78	-	0.15	-
CNT0.45	527.34	295.31	386.72	2.66	2.38	-	0.45	-
CNT0.05NS0.2	527.34	295.31	386.72	2.66	0.27	1.05	0.05	0.2
CNT0.15NS0.2	527.34	295.31	386.72	2.66	0.78	1.05	0.15	0.2
CNT0.45NS0.2	527.34	295.31	386.72	2.66	2.38	1.05	0.45	0.2
CNT0.05NS1.0	527.34	295.31	386.72	2.66	0.27	5.27	0.05	1.0
CNT0.15NS1.0	527.34	295.31	386.72	2.66	0.78	5.27	0.15	1.0
CNT0.45NS1.0	527.34	295.31	386.72	2.66	2.38	5.27	0.45	1.0

## Data Availability

Data are contained within the article.
